# Cryofouling avoidance in the Antarctic scallop *Adamussium colbecki*

**DOI:** 10.1038/s42003-022-03023-6

**Published:** 2022-01-21

**Authors:** William S. Y. Wong, Lukas Hauer, Paul A. Cziko, Konrad Meister

**Affiliations:** 1 Max Planck Institute for Polymer Research, 55128 Mainz, Germany; 2 Institute of Ecology and Evolution, University of Oregon, Eugene, OR 97403 USA; 3 University of Alaska Southeast, Juneau, AK 99801 USA

**Keywords:** Biooceanography, Biomaterials, Behavioural ecology

## Abstract

The presence of supercooled water in polar regions causes anchor ice to grow on submerged objects, generating costly problems for engineered materials and life-endangering risks for benthic communities. The factors driving underwater ice accretion are poorly understood, and passive prevention mechanisms remain unknown. Here we report that the Antarctic scallop *Adamussium colbecki* appears to remain ice-free in shallow Antarctic marine environments where underwater ice growth is prevalent. In contrast, scallops colonized by bush sponges in the same microhabitat grow ice and are removed from the population. Characterization of the Antarctic scallop shells revealed a hierarchical micro-ridge structure with sub-micron nano-ridges which promotes directed icing. This concentrates the formation of ice on the growth rings while leaving the regions in between free of ice, and appears to reduce ice-to-shell adhesion when compared to temperate species that do not possess highly ordered surface structures. The ability to control the formation of ice may enable passive underwater anti-icing protection, with the removal of ice possibly facilitated by ocean currents or scallop movements. We term this behavior cryofouling avoidance. We posit that the evolution of natural anti-icing structures is a key trait for the survival of Antarctic scallops in anchor ice zones.

## Abstract (web summary)

Wong et al report that the Antarctic scallop *Adamussium colbecki* appears to remain ice-free in shallow Antarctic marine environments where underwater ice growth is prevalent. They find that micro-ridge structures in the shell promote directed icing that may prevent the dangerous accumulation and firm attachment of buoyant ice.

## Introduction

The unwanted interaction with ice in our natural environments presents challenges with undesirable consequences: expansion or growth of frost/ice can impact power generation (solar panels, hydroelectric dams, and power lines), damage infrastructure (roads, railways, wind turbines), or be lethal to organisms^1–3^. Currently, growing efforts have focused on anti-icing surfaces developed for in-air environments^1,4–7^ to avoid, repel or retard ice formation. Promising strategies employ either micro-/nano-textured or very smooth liquid interfaces^8–10^ to encourage the shedding of accreted ice. Specific topological modifications have also been fabricated that redirect ice growth to only a subset of the surface, potentially reducing overall adhesive forces^11^.

In contrast to in-air environments, the driving factors of ice accretion on underwater surfaces^12–14^ are not understood, and mechanisms for its prevention remain unknown. Presently lacking a suitable terminology, we define the term “cryofouling” to describe the unwanted accumulation of ice on submerged surfaces. Naturally-occurring surfaces that avoid cryofouling may already exist, given that various cold-blooded organisms survive^15,16^ in the shallow marine environments surrounding Antarctica where cryofouling is prevalent and often hazardous to aquatic life^17,18^.

**Fig. 1:**
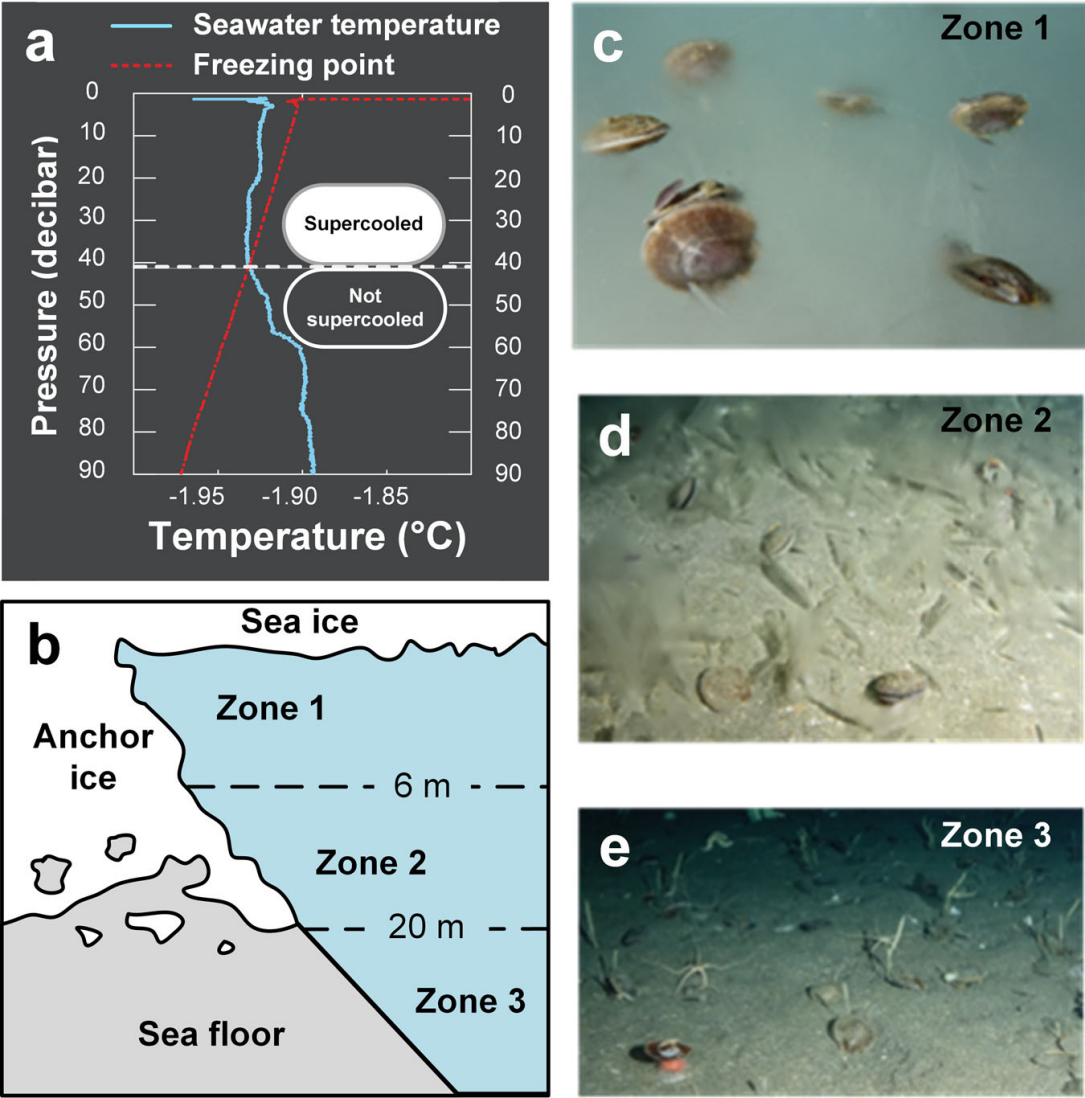
Antarctic scallops inhabit anchor-ice zones in Antarctica yet appear to remain ice-free. **a** Typical vertical profile of seawater conditions in austral spring in McMurdo Sound, Antarctica (14th Nov, 2012) showing seawater supercooling. Seawater supercooling drives anchor-ice growth, the prevalence, and thickness of which typically increases towards shallower depths. **b** Schematic representation of the anchor-ice zones in Explorer’s Cove, McMurdo Sound, Antarctica, informally defined herein using the definitions established by Dayton et al.^26^. **c**–**e** In Explorer’s Cove, Antarctic scallops occur in all zones, including the heavily anchor ice impacted shallowest areas (Zone 1, **c**) where they are found atop the growing anchor-ice blanket. Despite the abundant ice accretion on rocks and sediment in Zones 1 and 2, cryofouling of the exterior of the scallops’ mineral shells was never observed.

Cryofouling of underwater objects can occur when water is supercooled^19^ below its expected salinity- and pressure-dependent equilibrium freezing point. In coastal Antarctica, supercooled waters are produced by the interaction of coastal waters with massive, thick floating ice shelves producing small, freely floating ice crystals in the water column^20,21^. In some high-Antarctic environments, near-surface ocean waters are regularly supercooled by ~0.01 °C for up to half of each year^22^. Seawater supercooling in Antarctica, albeit minimal in magnitude, may drive substantial volumes of ice accretion on submerged surfaces^18^. For example, in McMurdo Sound (78 °S latitude), the supercooled conditions (Fig. 1a) that occur from ~July to December of each year cause the growth of a thick blanket of semi-consolidated ice crystals (up to 2–3 m) on the rocks and sediment of the shallow seabed (≲33 m depth), termed anchor ice^17,23^. Man-made materials such as oceanographic instruments, cables, plastic lines, and other equipment deployed into the supercooled water often experience rapid cryofouling^24^.

The formation of anchor ice (Fig. 1b), a form of cryofouling, has been proposed to occur either by the accumulation of suspended adhesive frazil ice particles, or by the in situ nucleation and growth of ice crystals on the host material^18,25^. Once formed, this underwater ice is stable as long as the temperature of the surrounding water remains at or below the freezing point, with additional growth favored by continued supercooling. As in the case of rocks and man-made materials, cold-blooded marine animals also risk cryofouling in supercooled seawater^18^. Diving researchers have documented that the diversity and abundance of benthic invertebrate species in McMurdo Sound, Antarctica are negatively correlated with the prevalence of anchor ice and/or supercooled seawater^17,18,26^. That is, in lacking evolutionary adaptations to counter the accretion of ice on their surfaces, most species are likely restricted to deeper areas, below the typical occurrence of anchor ice.

In shallower areas where anchor ice is common, the cryofouling of organisms can interfere with behavioral or biological processes, or remove individuals from the seabed given the buoyancy of accreted ice^18^. In the well-studied areas of southeastern McMurdo Sound, only a few species^21^ (e.g., the sea urchin *Sterechinus neumayeri*; the sea star *Odontaster validus* or the anemone *Isotealia antarctica*), presumably having evolved or serendipitously possessing characteristics that permit them to avoid cryofouling, survive and thrive in the shallow anchor-ice zone.

Here, we investigate how *Adamussium colbecki*, the Antarctic scallop, avoids cryofouling of the exterior surfaces of its mineral shells. Using a comparative approach, we compare the results of ice growth and adhesion experiments on Antarctic scallop shells to those performed on two temperate Atlantic scallop species that do not encounter ice in their habitats. Based on results of molecular phylogenetic analyses, *Placopecten magellanicus* (sea scallop) belongs to the same, small subclade as the Antarctic scallop, while the *Argopecten irradians* (bay scallop) is more divergent (Supplementary Fig. 1). We posit that cryofouling avoidance is a key trait permitting the survival of the Antarctic scallop in the anchor-ice zones of Antarctica.

## Results and discussion

The Antarctic scallop *Adamussium colbecki* is one of the few species of benthic organisms that we routinely observed in the icy upper reaches of the shallow anchor-ice zone in Explorer’s Cove, in western McMurdo Sound, Antarctica (Supplementary Movie 1). They inhabit both the deep, ice-free zones and the shallowest, iciest areas^27^ where they can even be found sitting atop the thick, growing anchor-ice blanket (Fig. 1c–e). The external surfaces of this benthic bivalve (hereafter referred to as “shell”) appear to remain ice-free, while rocks and other inanimate materials in the scallops’ vicinity are completely covered with ice (Supplementary Movie 1). In possessing an inanimate calcitic shell, this species may provide a unique example of a marine invertebrate that avoids cryofouling by truly passive means.

Similar to the well-studied locales in southeastern McMurdo Sound^17,26^, diver observations in Explorer’s Cove (2015, 2017, 2018, by PAC) revealed that the prevalence and thickness of the anchor-ice formations are highest in the shallowest locales and decreased with increasing depth of the seabed. From the underside of the surface sea ice (~2–3 m) to about 6 m depth, anchor ice formed a 60-cm-thick mat, covering greater than 90% of the seabed (Zone 1, Fig. 1c). From ~6 to 20 m depth, the anchor-ice cover was patchier, appearing to cover only 30–50% of the seabed (Zone 2; Fig. 1d). Anchor ice was never observed at depths below ~20 m (Zone 3; Fig. 1e). This depth may demarcate the maximum depth of supercooling occurrence in the study area (Fig. 1b). Live scallops were found to be distributed in all three zones. At the shallowest depths, aggregations of scallops were common even atop the growing anchor-ice blanket (Fig. 1c and Supplementary Movie 1). Interestingly, the shells of live scallops were never observed to be cryofouled during *~*30 research dives completed during the peak anchor-ice growth periods in Austral spring (October to December of 2015, 2017, and 2018), nor did any scallops appear to be frozen to the actively growing anchor-ice matrix on which they sat.

**Fig. 2:**
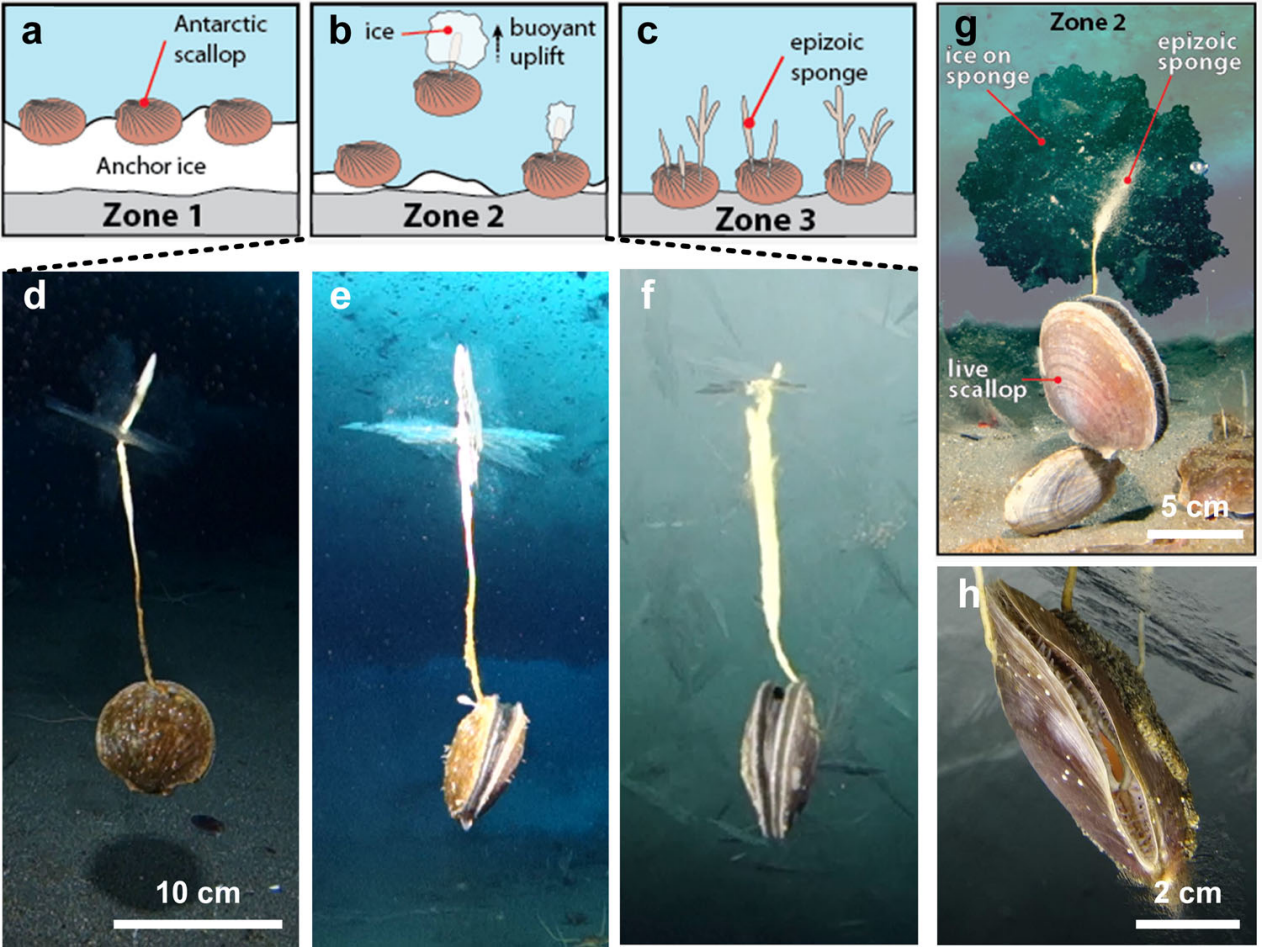
Sea sponge-colonized Antarctic scallops demonstrate the potential dangers for organisms that do not avoid cryofouling. **a**–**c** Unless colonized by the cryofouling-susceptible bush sponge (*H. balfourensis*), Antarctic scallops appear to be unaffected by cryofouling, even in areas where underwater ice growth is prevalent (Zones 1 and 2). **d**–**h** Photographic images depicting the progression of cryofouling-induced uplift (by buoyant ice) of sponge-colonized scallops in Zone 2. The negative consequence of cryofouling for scallops becomes apparent only when its surfaces have been colonized by the bush sponge *H. balfourensis*, which readily accrete ice. **g** When a sufficient volume of ice has accreted on the sponge, both species are rafted to the underside of the sea ice by buoyant flotation, where both appear to freeze in and die. Scallop is freezing into the underside of the sea ice. Scallop shell exteriors appear to be free of icing even after arriving at the underside of the sea ice. Note: Scallops arriving and freezing into the underside of the sea ice will eventually be enveloped by further sea ice growth. Scale bars indicated are approximates.

Demonstrating the potential dangers of cryofouling for Antarctic scallops is non-trivial because the scallops themselves appear to naturally avoid cryofouling. However, on numerous occasions, PAC documented cryofouling on bush sponges (*Homaxinella balfourensis*) that had colonized the external shell surfaces of Antarctic scallops. In these observations, the growth of ice^24^ on the epizoic sponges caused involuntary flotation of the host scallops due to the buoyancy^28^ of the ice attached to the sponge (Supplementary Movie 2). This resulted in the transport of both species to the underside of the growing sea-ice cover above, where they presumably froze in and died (Fig. 2a–f). Strikingly, the shells of cryofouled sponge-colonized scallops themselves, as well as uncolonized scallops in their immediate vicinity, appeared to remain devoid of ice. These observations suggest that the scallops themselves must be cryofouling-avoidant compared to the sponge, as well as to non-biogenic minerals in their vicinity (rocks, sand, and fine sediment) (Supplementary Movie 2).

Cryofouling of Antarctic scallops could potentially be deleterious in multiple ways beyond that resulting in buoyant uplift and relatively rapid death. For example, ice adhered to a scallop’s shell could impact their swimming and escape behaviors or occlude water flow paths necessary for filter-feeding. In this case, if the Antarctic scallops do not possess anti-cryofouling capabilities, a larger proportion of the population may likely suffer from the same hazards posed by icing to epizoic sponges: ice growth, involuntary flotation, and likely death when merged into the sea ice. Given that no observations of a cryofouled live scallop exist, the icing process on scallops may be different, posing lower risks for accumulation and flotation. The negative consequences on both feeding and motility functionalities (Fig. 2c–h) associated with cryofouling suggest that for organisms inhabiting areas of supercooled seawater, the selection pressure to evolve passive or active anti-icing strategies could be relatively strong.

**Fig. 3:**
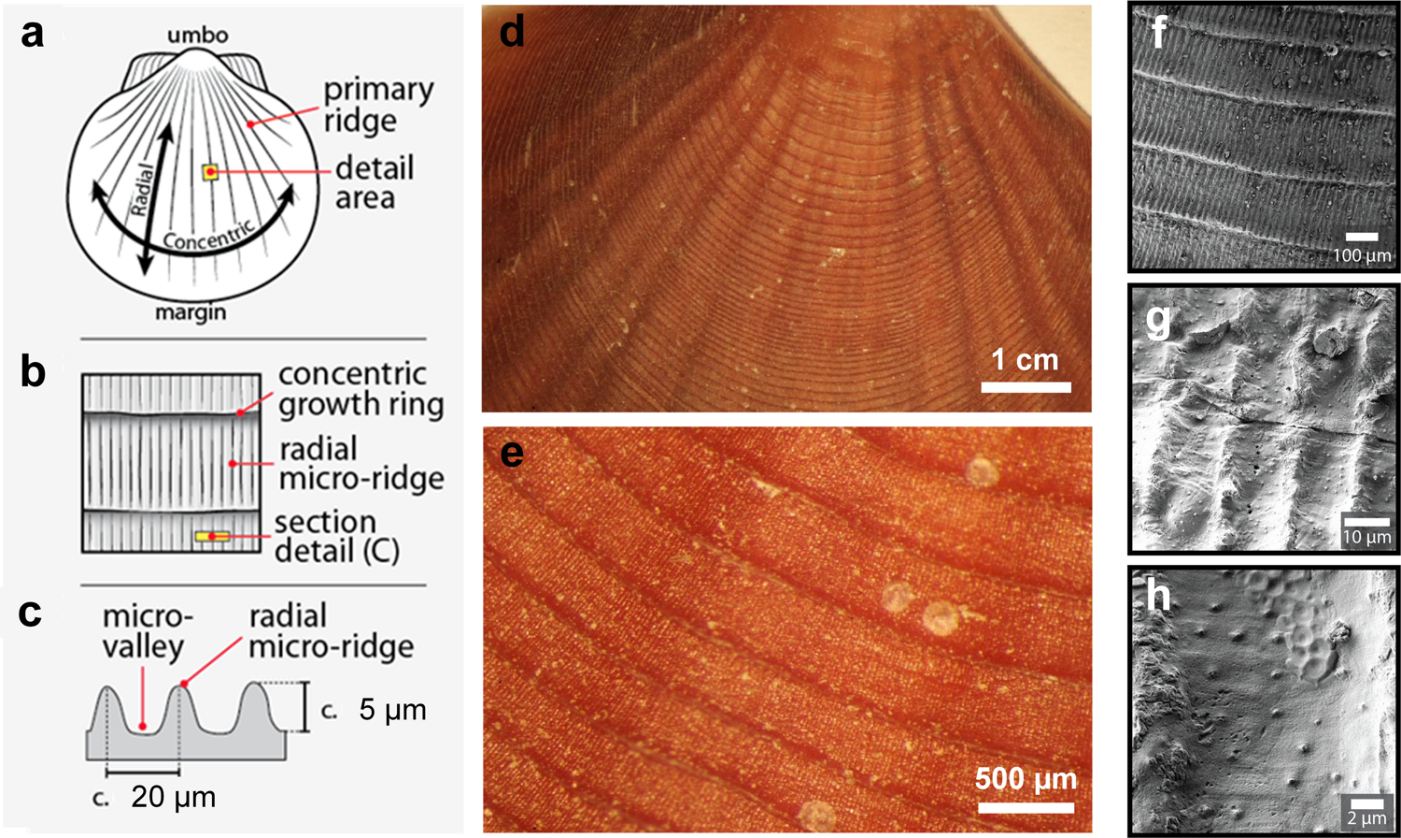
Surface characteristics of the Antarctic scallop’s shell features. **a**–**c** Terminology and schematics of features on the shell surface, **a**, **b** in-plane, and **c** section view. **d**, **e** Macroscopic images in visible light of the Antarctic scallop’s shell surface without magnification. The shell is covered by a thin, proteinaceous covering (periostracum), under which the calcitic material lies throughout the thickness of the shell. Radial rounded, primary ridges, and concentric growth rings are visible. **f**–**h** Scanning electron micrographs showing increasing magnification. **f** Concentric growth rings separating repeating series of **g** radial micro-ridges (c. 20 µm peak-to-peak). **h** Small protuberances are irregularly dispersed throughout the radial micro-valleys (Supplementary Fig. 3). Shells are oriented with the umbo at the top in all panels, except **c**. Shells of temperate control species are substantially rougher and less ordered than those of the Antarctic scallop (Supplementary Fig. 2).

The ability of surfaces to avoid in-air icing is influenced by properties like micro-structuring, surface roughness, hydrophobicity, chemical composition, or specific topological patterns^4,5^. Figure 3a–e shows the morphological characterization of the shells of the Antarctic scallop. In contrast to two non-Antarctic control species (bay scallop and sea scallop), the shells of the Antarctic scallop are macroscopically smooth, with only minute, elevated concentric growth rings and gently undulated primary ridges apparent to the naked eye. Scanning electron microscopy (SEM) images of the Antarctic scallop shells revealed a highly structured surface^29^ with concentric growth rings that separate micro-ridges and micro-valleys, forming a distinctive, regular hierarchical texture (Fig. 3f–h). That is, the areas between growth rings are defined by a series of relatively uniform radial micro-ridges. The shell surfaces of the two control species did not exhibit any ordered or repeating microstructures (Supplementary Figs. 2 and 3). For the Antarctic scallop, the spacing between concentric growth rings and radial micro-ridges along a line from the umbo to the margin were determined to be ~250 and ~10 µm wide, respectively. These features are not strictly uniform over the shell and may vary up to an order of magnitude, with the spacing of features increasing as a function of distance from the umbo. The microscopic surface features of larger (older) shells were slightly abraded, with surface wear appearing to increase towards the umbo–the oldest portion of the shell.

Next, we determined the elemental composition and roughness at different positions on the shell surface. Energy-dispersive X-ray spectroscopy revealed that the shell surface predominately consists of oxygen, carbon, calcium, traces of silicon, sodium, aluminum, and magnesium (Supplementary Fig. 4). These findings are consistent with a typical calcitic shell (CaCO_3_) laminated with proteinaceous periostracum. Surface composition was found to be identical at different locations of the analyses, including along concentric growth rings, micro-ridges, or the micro-valleys in between. The surface roughness was determined using atomic force microscopy (AFM) and revealed that the peaks of the concentric growth rings were the roughest (RMS roughness of 135 ± 57 nm), followed by the radial micro-ridges (RMS roughness of 96 ± 3 nm). In contrast, valley floors between these micro-ridges were relatively smooth (RMS roughness of 38 ± 9 nm), interrupted only by small protuberances (nano-grains, diameter of 83 ± 26 nm) that were irregularly dispersed throughout the radial micro-valleys.

**Fig. 4:**
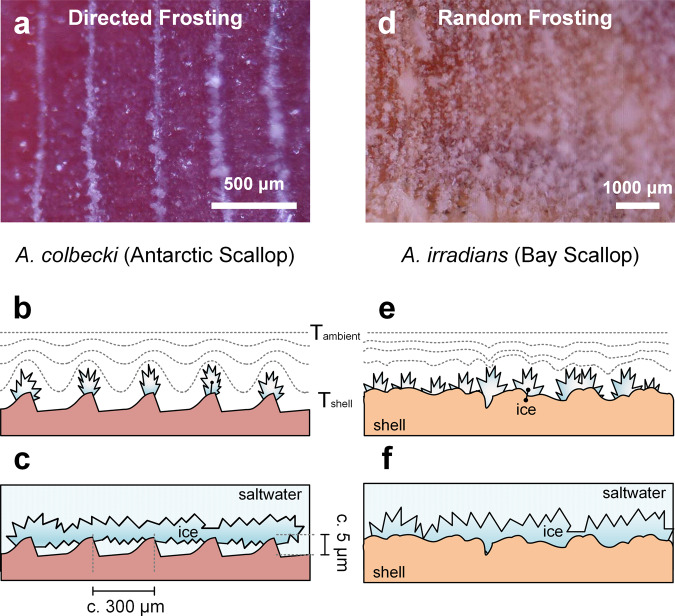
In-air frosting of scallop shells. The progression of ice accumulation was observed for shells placed atop a cold source in a temperature- and humidity-controlled chamber (20 °C, 60% relative humidity). **a** The Antarctic scallop preferentially directed ice nucleation and subsequent growth to the shell’s growth rings, termed directed frosting. **b** This patterned ice accumulation may also apply to ice growth in underwater environments, where it could reduce overall ice-shell contact area (**c**). **d** Directed frosting was not observed in control species (e.g., Bay scallop), where ice accreted in a patchy or uniform fashion over the entire surface (**e**). Likewise, such random but homogenously distributed nucleation-growth behaviors are likely to apply for ice growth in underwater environments, with continuous ice mats over the surface of the shell establishing higher overall contact adhesion force (**f**).

In nature, unwanted ice accretion on surfaces can occur either by the accumulation of suspended adhesive frazil ice particles or by in situ initiation of ice growth (heterogeneous ice nucleation) on a surface^18,25^. To test the possibility of preferential ice nucleation, the scallops were subjected to ice nucleation measurements and an in-air frosting assay. Figure 4a, d shows the results of in-air frosting experiments performed using a custom-built apparatus^30,31^ in an air-filled climate-controlled (20 °C) chamber at controlled humidity (60%). Compared to the temperate control species, ice nucleation on the Antarctic scallop shell occurred later and subsequent ice growth was directed toward specific surface features (Fig. 4a, b and Supplementary Movie 3). Ice appears to accumulate only on the growth rings, leaving ice-free micro-ridges. As the experiment progressed, ice that had nucleated on the growth rings continued to grow vertically, thereby preventing further imaging of the ice-shell interface (Supplementary Movie 3). We observed no inter-ring bridging of ice and nucleated ice crystals grew preferentially upwards while enlarging in size. In contrast, neither control species showed any obvious signs of directed ice nucleation and appeared to frost uniformly over the surface (Fig. 4d, e). These results indicate that the Antarctic scallop may possess an ability to control ice nucleation and directed ice growth on its shell surfaces.

For the Antarctic scallop, the mechanism of directed frosting occurs over two sequential steps: (1) initial condensation-frosting on growth rings, followed by (2) continuous frost growth. (1) The presence of sharp/rough edges (such as those on the growth rings), is known to enhance liquid nucleation by reducing the energy barrier^32^. Since more liquid water resides at the growth rings, here the nucleation of microscopic ice is more likely^33^. (2) Upon the formation of these microscopic ice domains as ice stripes (on growth rings), the vapor field around the ridges becomes compressed. The compressed vapor field generates locally steeper vapor gradients and thus stronger vapor fluxes towards the ice on the ridges (Fig. 4b). As a consequence, ice growth on the stripes is enhanced compared to the valleys. The vapor transport in the air during frosting is analogous to the heat transport underwater during icing. At the ridges, the latent heat generated is removed more quickly, facilitating accelerated growth^34,35^. Furthermore, since the vapor pressure of ice is lower than that of water^36^, the condensed water in the micro-valleys would be redirected to the ridges. This process would result in the micro-valleys being left comparatively free of ice, per Fig. 4a.

A caveat remains, as the shell surface temperature during frosting assays (in-air) is significantly lower (c. −10 to −15 °C) than in nature. In its natural habitat, the lowest temperatures experienced by surfaces immersed in seawater (Fig. 4c, f) are rarely much below the equilibrium freezing point (~−1.9 °C), thus making heterogeneous ice nucleation on shells unlikely. However, the ability to direct ice growth may influence the probability of frazil ice adhering and anchoring strongly to the shell surface, thus facilitating easier removal through mechanical or passive environmental forces.

We performed ice-adhesion measurements to test whether the apparent cryofouling avoidance of the Antarctic scallop’s shell in nature could arise from reduced adhesion forces between adhered ice and the shell’s surface. If adhesion is sufficiently low, ice could detach and float away under behavioral (movements, locomotion^37^) and/or environmentally induced forces (physical interactions, water currents, buoyancy of ice), or a combination of the above. This is particularly relevant once a sufficiently high ratio of ice volume to attachment surface area has been achieved. To provide complementary insight, both in-air and underwater ice-adhesion measurements were performed.

**Fig. 5:**
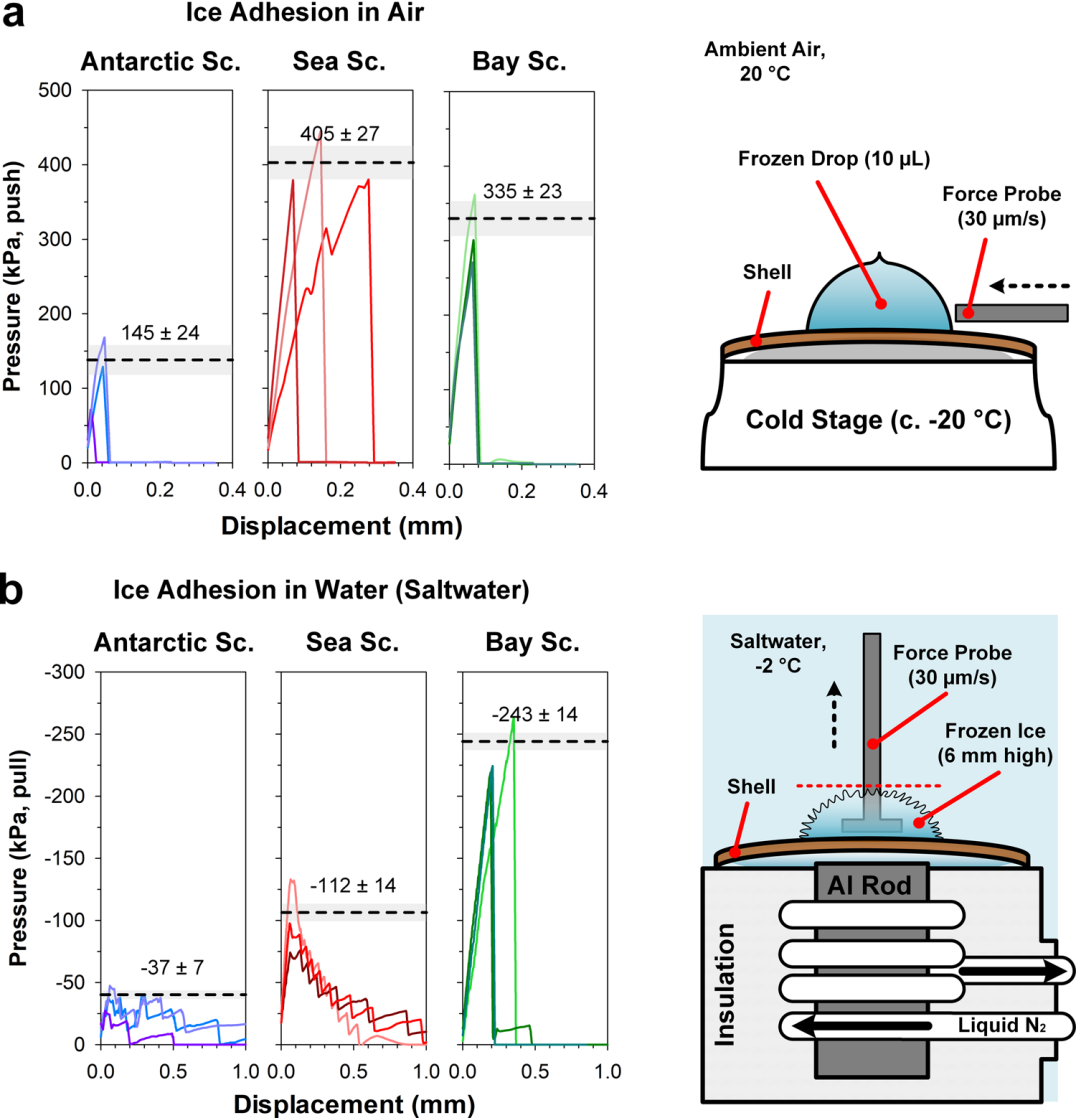
Ice-adhesion measurements for Antarctic, Sea, and Bay scallops. **a** Displacement (lateral) of ice drops (10 µL) from the surface of shells in a humidity-controlled chamber. Shell temperature: −10 to −15 °C (thickness-dependent). Peak force was achieved immediately before the complete detachment of the drop from the surface. **b** Displacement (normal) of accreted ice grown in simulated seawater at its freezing point (35 g/L NaCl, c. −2 °C). Mean peak recorded force (dashed lines) ±1 SE (shaded areas) for three repetitions of each experiment are shown. Experimental details are available in the Supplementary Methods and Materials.

The in-air ice-adhesion strength was determined^30^ by laterally shearing off drops of frozen freshwater from target scallop shells (Supplementary Movie 4). The recorded force curves show that ice adheres less strongly to the Antarctic scallop compared to the control species, the sea and bay scallops, as shown in Fig. 5a. For the Antarctic scallop, the in-air ice-adhesion strength was 145 ± 24 kPa (mean ± SE), ~2–3 times lower than both control species, with adhesion strengths of 335 ± 23 and 405 ± 27 kPa for the sea scallop and bay scallop respectively (Fig. 5a). We further observed that when the frozen drops sheared off the Antarctic scallop, the ice-shell fracture interface exhibited distinct growth ring-patterned fracture lines. In contrast, frozen drops detached from the control species appeared to have a uniform fracture interface (Supplementary Fig. 5 and Supplementary Movie 5). This observation could be explained by the incomplete contact adhesion of the frozen drops to the Antarctic scallop’s shell due to the spacing of small repetitive structural elements on the shell’s surface. As such, the overall area of ice-shell adhesion is reduced, thereby lowering the overall effective adhesion force.

The strength of ice adhesion (N/m^2^ or Pa) to shells underwater was determined using a custom-built apparatus. The apparatus determined the forces required for ice detachment when adhered ice was pulled perpendicular to and away from the shell surface. Underwater ice-adhesion experiments were performed in saltwater (35 g/L NaCl), simulating the seawater conditions in Antarctica. A cold source beneath a small section of the shell induced ice growth up to 6 mm in height above the shell surface, (Supplementary Movie 6), thereby encasing a perforated titanium plate (1 cm square, 4 × 2 mm-diameter holes) which was in turn connected to the force probe. The probe was then drawn upwards (normal to the shell, at 30 µm/s, Supplementary Movie 7). As for experiments in air, the recorded force curves in underwater experiments reveal that ice adheres weakly to the Antarctic scallop shell as compared to those of the two control species (Fig. 5b and Supplementary Movie 8). The Antarctic scallop experiences up to 3 times lower ice adhesion (37 ± 7 kPa, mean ± SE) than the sea scallop (112 ± 14 kPa) and more than 6 times lower adhesion than the bay scallop (243 ± 14 kPa) (Fig. 5b). At 37 ± 7 kPa, the Antarctic scallop’s shell shows underwater ice adhesion (normal to the surface) comparable to industrially-developed in-air anti-icing surfaces (1–50 kPa)^4^. However, if attachment forces in nature approach this value in a static environment (i.e., no water currents or scallop swimming behaviors), ≥3.7 cm^3^ of ice would need to be attached per mm^2^ of ice-shell contact area for the passive removal of ice under its own buoyant forces. Therefore, the “passive” removal of ice from Antarctic scallops must result from a combination of surface-to-environment factors. That is, the removal of ice crystals with small contact areas, such as platelet ice with its fine dendritic structure, may be facilitated by drag forces arising in natural water currents or induced by the rapid opening and closing of the scallop’s valves during swimming behaviors^37^. In these cases, adherent ice on the microstructures of the shell would likely experience the initiation of cracks that would subsequently facilitate ice removal given water movements or other physical disturbances.

Whether the cryofouling-avoidant surfaces of the Antarctic scallop’s shell arose under evolutionary selection pressure remains unclear. Based on fossil records, the exclusively Antarctic scallop genus *Adamussium* first appeared in the early Oligocene, some 33 million years ago (mya)^38,39^. This time period roughly coincides with the onset of the major glaciation of Antarctica (c. 35 mya). The Antarctic scallop, *Adamussium colbecki*, appears to have arisen more recently, in the late Pliocene (<5 mya), and remains the only extant scallop species in Antarctica^38,39^. Given that fossils of other, earlier members of the genus also appear to display similar surface microstructure^38,39^, this functional micro-ornamentation could conceivably be a lineage-specific adaptive trait that evolved in response to the threat posed by the presence of underwater ice formation in Antarctica. In the alternative, the cryofouling avoidance observed for the extant *Adamussium colbecki* may simply be a serendipitous outcome of pre-existing shell microstructure that evolved under unrelated developmental and environmental selection pressures. Regardless of the origin of the trait, the anti-cryofouling characteristics are clearly important for the survival of the Antarctic scallop today, at least within the shallow anchor-ice zone of coastal Antarctica. Establishing the biological relevance, ecological impact and the evolutionary timeline of the Antarctic scallop’s anti-cryofouling capabilities merit additional investigations, as they are beyond the scope of this work.

## Conclusion

For decades, ecologists have considered anchor ice to be an important agent of disturbance in the shallow-water benthic communities of McMurdo Sound, Antarctica. However, the mechanisms underlying the differential effects of underwater ice growth among species remain unexplained. Our observations of Antarctic scallops in their natural environment suggest that their exposed mineral shells passively avoid cryofouling, even in benthic habitats impacted by sustained underwater ice production. Characterization of these scallops’ shells revealed the presence of a unique distribution of growth rings and micro-ridges, with icing experiments suggesting that these structures modify the growth and/or adhesion of ice to the shell surface. In this model, the concentric growth rings promote directed icing events, leaving micro-ridges and valleys (i.e., the bulk of the shell’s surface area) ice-free. This directed ice growth behavior would result in the formation of fragmented ice crystals having large grain boundaries, thus helping to prevent the dangerous accumulation and firm attachment of buoyant ice that could lead to death or otherwise impact normal scallop behaviors. This specialized shell surface phenotype was not observed in two scallop species from temperate climates, in which ice-adhesion strength was determined to be markedly higher. Together, our observations support the inference that the Antarctic scallop’s possession of a cryofouling-avoidant shell surface, together with environmental and/or behavioral factors, this could be a key trait contributing to the species’ success in icy, shallow nearshore habitats of Antarctica. These findings provide a tentative step towards a better understanding of the complex interaction of polar benthic invertebrates with the intriguing phenomenon of underwater ice growth in supercooled water.

## Methods

### Scallop samples

Live *Adamussium colecki* (Antarctic scallop) were collected from New Harbor, Antarctica by SCUBA divers in November 2015. Temperate control species, including *Agropecten irradians* (bay scallop) and the *Placopecten magellanicus* (sea scallop) were obtained from the Marine Biological Laboratory, collected in the Atlantic Ocean in the vicinity of Woods Hole, MA, USA. Shells of all species were opened, cleaned of adherent body tissues, thoroughly rinsed in fresh deionized water, and then allowed to dry at room temperature prior to their use in experiments or analyses.

### Microscopic and surface analysis

The exterior surfaces of Antarctic scallop valves were analyzed using scanning electron microscopy (SEM; Zeiss, LEO 1530 Gemini). Atomic force microscopy (AFM) was used to provide nanometer-resolution mapping (300 kHz, tapping mode) of surface geometries and surface roughness (4-µm^2^ scan area) on the Antarctic scallop. The surface elemental composition and its variation across the exterior valve surface were determined using energy-dispersive X-ray spectroscopy (EDS) for Ca, C, O, F, Na, Mg, Al, Si, and Ca. This was performed on the growth rings, micro-/nano-ridges, and micro-valleys.

### Ice-adhesion (in air and underwater) analysis

In-air frosting was performed using a custom apparatus in an air-filled climate-controlled (20 °C) chamber at a controlled humidity (about 60% relative humidity; Supplementary Fig. 6). Ice-adhesion strength of ice-on-valve in air was determined for drops of frozen freshwater which were then sheared off by a lateral force. A force sensor (PCE-DFG N 20, PCE Instruments GmbH, recording at 200 Hz) attached to a probe was engaged, moving at c. 30 µm/s via a motorized stage (Thorlabs). The probe contacted ice drops at c. 0.5 mm above the ice-shell interface, resulting in a rise in measured force. The lateral motion of the force probe eventually broke the ice drop off the surface, resulting in a rapid drop in the force measured (Supplementary Fig. 6). Underwater ice adhesion on valves was determined using a custom-built apparatus (Supplementary Figs. 6–8). The apparatus determines the forces required for ice detachment when adhered ice was pulled perpendicularly away from the frozen shells. In this case, shells were mounted in a tank (10 L) containing a simulated Antarctic seawater environment (35 g/L laboratory-grade NaCl in deionized H_2_O, maintained at −2° ± 0.2 °C). A perforated aluminum plate (1 cm square, 1 mm thick, with four 2 mm-diameter equidistant perforations) was mounted on the end of a force probe. The ice-adhesion strength (N/m^2^ or Pa) of the adhered ice was determined by retracting the force probe vertically, normal to and away from the valve surface (30 µm/s) using a motorized stage until the ice detached from the shell surface.

### Statistics and reproducibility

All experiments were performed at least three times and the reported error bars are standard errors.

### Reporting summary

Further information on research design is available in the Nature Research Reporting Summary linked to this article.

## Supplementary information

Supplementary Information

Description of Additional Supplementary Files

Supplementary Movie 1

Supplementary Movie 2

Supplementary Movie 3

Supplementary Movie 4

Supplementary Movie 5

Supplementary Movie 6

Supplementary Movie 7

Supplementary Movie 8

Reporting Summary

## Data Availability

All data are available from the corresponding authors on reasonable request.
