# Supplementary material for: Cryofouling avoidance in the Antarctic scallop Adamussium colbecki
Source: Commun Biol. 2022 Jan 21;5:83. doi: 10.1038/s42003-022-03023-6 (PMC8783024; doi:10.1038/s42003-022-03023-6)
Supplement: Supplementary file 1 — Supplementary Information [file 42003_2022_3023_MOESM1_ESM.pdf]

Supplementary Materials for

Cryofouling Avoidance in the Antarctic Scallop *Adamussium colbecki*

William S. Y. Wong[1†], Lukas Hauer[1†], Paul A. Cziko[2]*, Konrad Meister[1,3]*

[1]Max Planck Institute for Polymer Research, 55128 Mainz, Germany
[2]Institute of Ecology and Evolution, University of Oregon, Eugene, OR, 97403, USA
[3]University of Alaska Southeast, Juneau, AK, 99801, USA

Keywords: Underwater fouling, Anti-icing surfaces, Cryofouling, Antarctic benthic marine ecology, Scallops

[†]denotes equal contribution
*corresponding author: pcziko@uoregon.edu and meisterk@mpip-mainz.mpg.de

This file includes:

Supplementary Methods and Materials
Supplementary Discussion
Supplementary Figures

Additional Supplementary Information includes: Supplementary Movies 1-8

Supplementary Methods and Materials

Collection and preparation of specimens

Juvenile and adult Antarctic scallops (c. 25 to 75 mm, umbo to edge) were collected live from New Harbor by SCUBA divers in November 2015. After opening and eviscerating the animals, the thin calcitic valves were separated (as half-shells) and soaked in fresh water to prevent salt deposits. Thereafter, the shells were dried in air, packaged separately in plastic bags and shipped to the respective institutions for analysis. Shells of freshly-collected temperate control species, including the subtidal bay scallop (*Agropecten irradians*) and the sea scallop (*Placopecten magellanicus*) were obtained from the Marine Biological Laboratory, collected in the Atlantic ocean in the vicinity of Woods Hole, MA, USA, and prepared similarly. Recent molecular phylogenies (Supplementary Figure 1) show that the sea scallop is closely related to the Antarctic scallop (*Placopecten magellanicus* and *Adamussium colbecki* respectively), while the bay scallop (*Agropecten irradians*) is more divergent (Supplementary Figure 1). Neither of the control species encounter ice in their habitats. All analyses were performed only on the exterior surfaces of the shells, without regard for which shell was being used ("left" or "right", typically top and bottom, respectively, given their normal orientation on the substrate in nature). This is the norm considering that surface characteristics were found to be similar on both shells of all species.

Surface structure and composition

Macroscopic observations

Shells of the Antarctic scallop are macroscopically smooth, with growth rings and micro-ridges that are observable at the millimetric-to-micrometric scale, extending out along the radial axis between growth rings. The exterior surfaces of shells are laminated with a reddish-colored, thin periostracum (proteinaceous shell covering), which is also alternately layered with calcitic material throughout the thickness of the shell (< 1 mm). In contrast, the surfaces of both control species (Bay and Sea scallops) are distinctively rougher. There are no observable packing of regularized ridges.

Scanning electron microscopy

The exterior surfaces of Antarctic scallop shells were analyzed using scanning electron microscopy (SEM; Zeiss, LEO 1530 Gemini). SEM analyses revealed radial rounded, primary ridges and concentric daily growth rings. The daily growth rings separate repeating series of radial micro-ridges orthogonal to the growth direction (c. 16 µm peak-to-peak). Surface features are consistent with previous descriptions. Control species (Sea and Bay Scallops) were also analyzed by SEM, but no distinctive self-repeating micro- or nano-structures were noted.

Dimensions of surface features were not entirely uniform over the surface of the shell. As the scallop ages, growth occurs at the margin, but the dimensions of micro-ridge surface features remain identical. Nonetheless, due to aging and surface erosion, we noted that

features may be less pronounced and shallower towards the margin of the shell. For the Antarctic scallop, we used SEM to measure the dimensions of features along a line from the umbo to the margin.

Atomic force microscopy

The ability of shell surfaces to avoid cryofouling may be due to the characteristics of surfaces at sub-microscopic scales, such as surface roughness or specific topological patterns. Atomic force microscopy (AFM) was used to provide nanometer-resolution mapping of surface geometries and surface roughness on the Antarctic scallop. Multiple scans were performed in the tapping mode, at a frequency of 300 kHz on the concentric growth rings' peak, the radial micro ridges and the valleys separating the ridges. Four distinct geometrical features were noted, including ring-peaks, micro-ridges, micro-valleys, and nano-grains each with a 4-μm^2 scan area. The analyses indicated that the ring-peaks were the most rough (rms roughness of 135 ± 57 nm), followed by the radial micro-ridges (rms roughness of 96 ± 3 nm). In contrast, valley floors between the small ridges were relatively smooth (rms roughness of 38 ± 9 nm), interrupted only by small protrusions (nano-grains, diameter of 83 ± 26 nm) that were irregularly dispersed throughout the radial micro-valleys. The variation in surface roughness over the surface could indicate that, should icing occur underwater, ice attachment may be enhanced for the roughest areas, *i.e.* on the concentric growth ring feature. This may result in heterogeneous ice attachment and growth that could facilitate facile removal.

Surface elemental composition

The cryofouling avoidance of the Antarctic scallop may also be attributed to the presence of unique molecular or elemental composition. To verify this potential parameter, energy-dispersive X-ray spectroscopy (EDS) was performed. The surface elemental composition and its variation across the exterior shell surface were determined for Ca, C, O, F, Na, Mg, Al, Si and Ca on a pristine, small shell from a juvenile scallop (c. 3 cm diameter). Larger adult specimens were not investigated due to the presence natural surface wear that may affect results. This technique provides information up to a micrometer in depth resolution.

EDS analyses of the Antarctic scallops' shell surfaces revealed consistent peaks in oxygen, carbon, calcium and traces of silicon, sodium, aluminum and magnesium. These findings are consistent with a typical calcitic shell (CaCO3) laminated with proteinaceous periostracum (Supplementary Figure 4). Surface composition was found to be practically identical regardless of the location of the analyses, including along ring-peaks, micro-ridges, and micro-valleys. The overall increased presence of O alongside inorganic metals could indicate the presence of oxides that could potentially increase the hydrophilicity of the surface. Traces amounts of F, together with C, could indicate that hydrophobic fluorocarbons were present. Unfortunately, fluorocarbons are almost always synthetic compounds and this likely resulted from contamination in the environment, packaging materials during shipping or exposure to lab conditions. Results from EDS analyses nevertheless suggest that any resistance to ice accretion is not a result of unique elemental composition that could provide a differential hydro- or ice-phobic surface patterns. Moreover, given that all surface features appeared to have similar elemental composition,

any differential resistance to cryofouling over the shell surface is instead likely due to differences in their microstructural characteristics (Supplementary Movie 8).

Surface wettability

The hydrophobicity of the Antarctic scallop and controls (Bay and Sea) scallops were estimated using measurements of dynamic contact angles (DCAs) of water drops (MilliQ) deposited on the surface in air. While the scallop is never exposed to air in nature, this analysis permits comparison of the relative hydrophobicity between species. Contact angles were measured for 3 drops (5 µL each) placed on the shell surfaces using the sessile drop method.[1] Contact angles between the droplets and the shells were measured on the shells continuously up to 40 s after contact, giving insight into the spreading dynamics and wetting equilibrium. It should be noted that contact angle measurements are almost always complicated by the macrostructure of the surface (*i.e.* curves). Nevertheless, results indicate that the surface is comparatively hydrophilic (CA of 55° to 80°), perhaps akin to the typical contact on polymethyl methacrylate (PMMA) or plexiglass.[2] Both controls and Antarctic scallops show similar wetting behaviors. The presence of proteinaceous periostracum covering the shell surface may have contributed to an increased hydrophobicity (*vs.* pure inorganic oxides). These results suggest that surface wettability plays a limited role in limiting ice adhesion.

Analyzing naturally-present ice nucleation / adhesion geometries (In-air frosting)

To test the possibility of preferential nucleation, control and Antarctic scallops were subjected to a dynamic in-air frosting assay. At the solid-air interface, surface-mediated ice nucleation increases as a function of surface roughness.[3] On a subfreezing shell surface in air, areas of preferential ice nucleation would demarcate areas of increased surface roughness. Conversely, a homogenous distribution of ice nucleation over the surface would indicate similar roughness along the entire surface. However, such a relationship has not been tested for surfaces submerged in supercooled water. As far as we know, surface-mediated nucleation remains unlikely at mild supercooling (< 1-2 °C). Therefore, a secondary mechanism may exist in Nature. In the natural environment, ice crystals pre-existing in seawater (frazil ice) may experience higher propensity to adhere to roughened portions of a surface as compared to smoother surfaces. Therefore, ice adhesion and subsequent growth at roughened areas may be more likely. Nonetheless, in an analogous manner, the in-air frosting assay may still provide a hint on where ice accretion may occur in an underwater environment.

In-air frosting was performed using a custom apparatus in an air-filled climate-controlled (20°C) chamber at a controlled humidity (about 60% relative humidity) (Supplementary Figure 6). The interior surface of shells were adhered to a cold stage (Linkam THMS-600 with LNP-96 controller) using thermal paste, before the cold stage was cooled (-50°C/min) and then held at -25°C for the duration of the experiment (500 s). Due to differences in thermal transfer across the shells of the three species having different thicknesses, shell surface temperatures ranged from -10° (bay and sea scallops) to -15°C (Antarctic scallop). An optical microscope permitted continual visual inspection of ice accretion on the surface

during the experiment (125x magnification for the Antarctic scallop, 36x for the other species). Ambient air was steady and no air currents were used to induce frosting.

Heterogenous nucleation of ice formation on shell surfaces (In-water icing)

In exceedingly pure water devoid of suitable ice-nucleating sites or pre-existing ice, energy barriers prevent the phase transition from water to ice. That is, spontaneous homogeneous nucleation of ice from bulk water requires temperatures to be substantially lower than the equilibrium freezing point (< -39°C).[4] However, the initiation of ice growth on an impurity in contact with or suspended in the water may occur at higher temperatures (heterogeneous nucleation of ice). Some surfaces, minerals and proteins are known to encourage the formation of ice a relatively low supercoolings, *i.e*, as high as only 2-4°C below the equilibrium freezing-melting point.[5,6] In air, where temperature extremes may far exceed those found in the ocean, surfaces with very low temperatures may directly induce nucleation. In contrast, the lowest temperatures experienced by surfaces immersed in natural seawater are never much lower than the equilibrium freezing point (c. -1.9°C at the surface. The maximum seawater supercooling observed in McMurdo Sound, Antarctica is typically much less than 0.1°C (Figure 1),[7] suggesting that cryofouling arises predominantly from the attachment and further growth of preexisting ice crystals suspended in the water column.

Strength of ice adhesion to shell surfaces

The cryofouling avoidance of the Antarctic shell may be secondarily attributed to reduced adhesion forces between the adhered ice and the shell surface. If adhesion is sufficiently low, adhered ice may detach and float away under environmental forces (locomotion, physical interactions, water currents, buoyancy of ice, or a combination of the above). This may take place once a sufficiently high ratio of ice volume to attachment surface area occurs. The ice adhesion strength of all shells were assessed in both the in-air and simulated seawater environment.

In-air ice adhesion

Ice adhesion strength of ice-on-shell in air was determined for drops of frozen freshwater which were then sheared off by a lateral force. These measurements are typically performed for so-termed anti-icing surfaces. Experiments were conducted within a larger climate control chamber that houses additional instrumentation (force sensor and guide rails). Shells are first mounted onto the cold stage using thermal paste. Shells were then immobilized (at -20 °C) by freezing excess freshwater around the target location until it is completely encased in ice. This prevents lateral movement or detachment from the stage during lateral adhesion force measurements. A stream of warm nitrogen gas was used to deice and dry the target location, before immediately depositing a drop of freshwater (10 μL) on the surface. The drop was then allowed to freeze. As the shells are irregular in nature, drops may spread somewhat prior to freezing, resulting in a variable contact area. The resulting contact area was measured by an optical microscope at a magnification of 26.8x, such that specific lateral adhesion forces could then be computed (N/m^2 or Pa). Thereafter, a force sensor recording at 200 Hz (PCE-DFG N 20, PCE Instruments GmbH),

attached to a probe was engaged, moving at 30 µm/s via a motorized stage (Thorlabs). The probe contacts the ice drop, resulting in a rise in measured force. The lateral motion of the force probe eventually breaks the ice drop off the surface, resulting in a rapid drop in the force measured (Supplementary Figure 6). Raw force data were smoothed using a 100-point moving average (*i.e.* a datapoint every 0.5 s) to filter out noise prior to analysis. Dynamic and peak forces were tabulated, with the latter achieved immediately prior to ice drop detachment.

Underwater ice adhesion (simulated seawater)

Underwater ice adhesion on shells was determined using a custom-built apparatus (Supplementary Figures 7-8). The apparatus determines the forces required for ice detachment when adhered ice was pulled perpendicular away from the frozen shells. This serves to improve understanding behind the indication of the forces necessary for de-icing of accreted ice from the shells under its own buoyancy or shear forces in moving water currents. In this case, shells are mounted in a tank (10 L) with simulated Antarctic seawater environment (35 g/L laboratory-grade NaCl in deionized H_2O, at -2° ± 0.2°C). A perforated aluminum plate (1 cm square, 1 mm thick, with four 2 mm-diameter equidistant perforations) was mounted on the end of a force probe. This probe was pushed (5 N) against a sample surface (parallel to the shell, << 1 mm separation). This maximizes contact before commencing icing. A thin layer of frozen fresh water (prior to immersion) was first frozen in place to ensure consistent heat transfer.

Heat transfer to a cooling element beneath the shell (contacting the internal shell surface) encourages localized ice to grow on a small area on the shell's external surface. This takes place within the order of 10 minutes (shell-dependent). Ice nuclei likely already exist in the simulated seawater as the cold source (LN2) is delivered from deeper in the tank, where ice has already been growing. The initial ice growth was therefore likely seeded by these pre-existing ice nuclei and not from direct heterogeneous nucleation on shells. With continued cooling, ice was allowed to encase the plate, rising up to a small dome of ice that extends 6 mm above the surface of the plate (determined by markings on the force probe, and monitored by an underwater camera). Temperature measurements at the edge of the growing ice recorded a value of between -3 °C to -5 °C, indicating an undercooling of between -1 to -3 °C below the freezing point (-2 °C). Note: Due to the localized cooling coming from below the shell and the larger undercooling, the experiment is only an approximation of actual natural supercooling conditions under which ice grows.

The ice adhesion strength (N/m^2 or Pa) of the adhered ice was determined by retracting the force probe vertically, normal to and away from the shell surface (30 µm/s) using a motorized stage. Dynamic forces, measured at 200 Hz increased until the ice-to-shell adhesion was broken. Raw force data were smoothed using a 100-point moving average (*i.e.* a datapoint every 0.5 s) to filter out noise prior to analysis. Peak forces recorded include contributions from breaking ice-surface adhesion plus fracturing ice at the periphery of the probe plate, though the latter is expected to be approximately equivalent for all surfaces.

Supplementary Figures

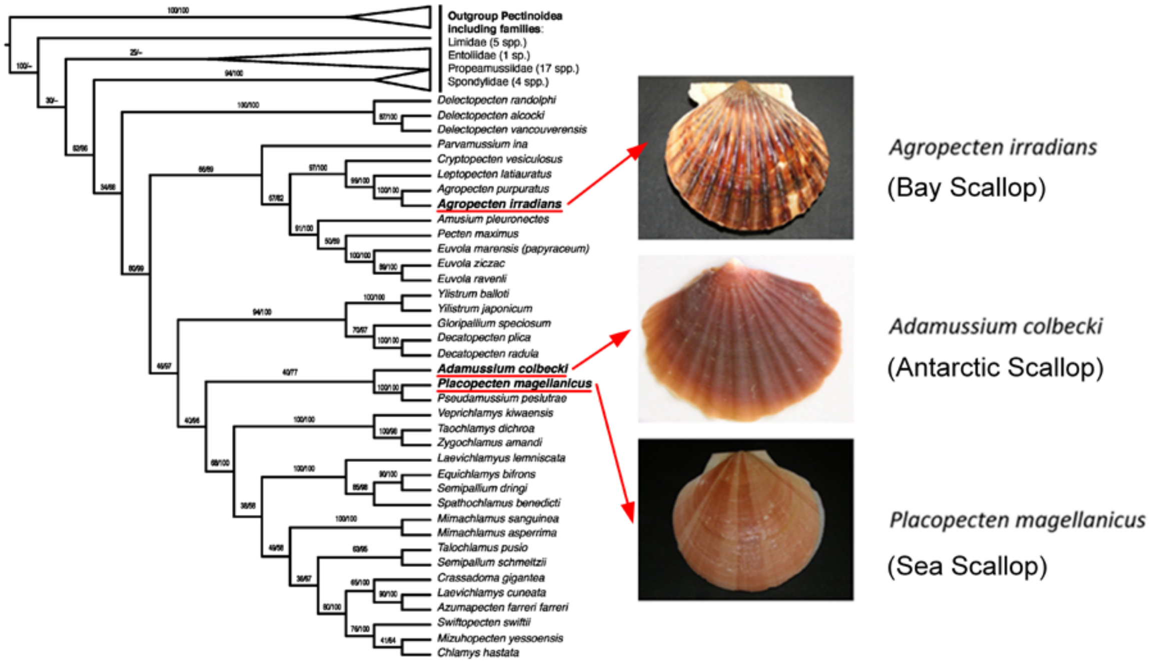

Supplementary Figure 1. Phylogenetic relationships between the tested scallop species. A recent maximum likelihood molecular phylogenetic analysis, based on multiple mitochondrial and nuclear genes, reveals the relationships between the tested scallop species. Members of the family Pectinidae, to which all three tested species belong, are represented in the expanded clade. The Antarctic scallop and the sea scallop belong to the same small subclade and are relatively closely related, while the bay scallop is more distantly related. The molecular phylogeny presented here is adapted from Dalton *et al*.[8] Bootstrap support values followed by Bayesian posterior probabilities are presented on each branch.

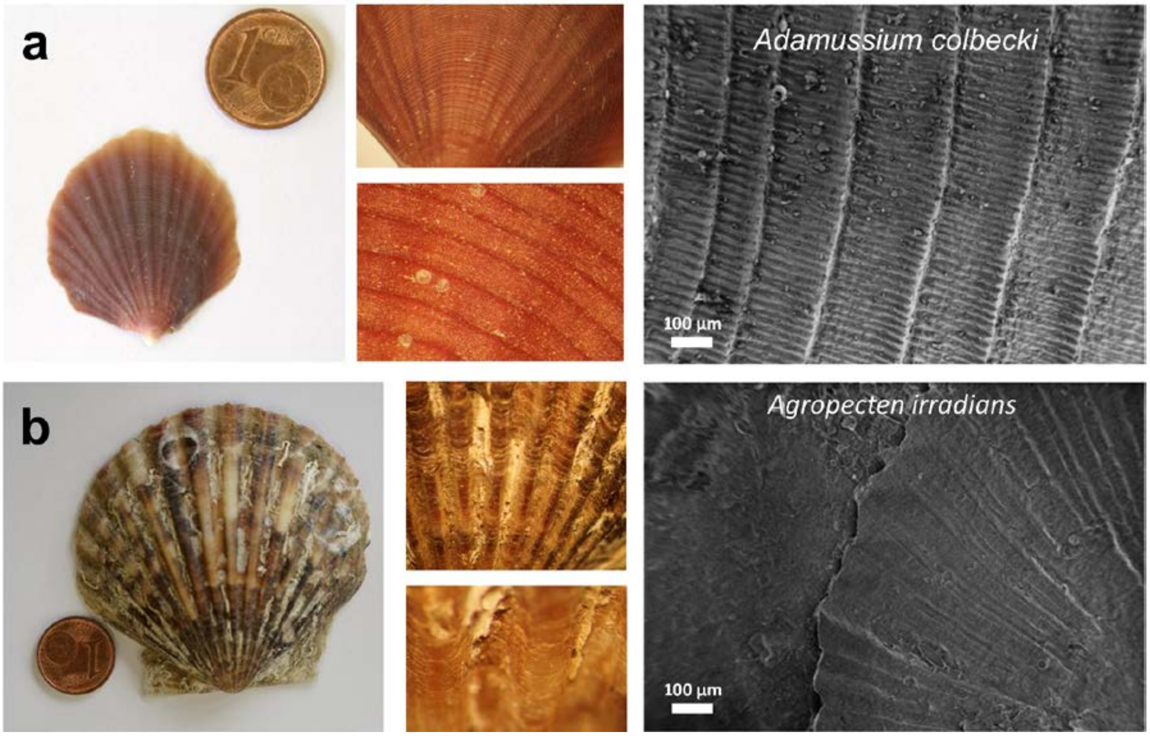

Supplementary Figure 2. Photographic imaging of the Antarctic and Bay scallop shell. a) *A. colbecki* species where regularized concentric growth rings and micro-ridges/valleys are clearly visible within scanning electron micrographs. b) *A. irradians* species where rough centimetric primary ridges that run from the umbo to the edge of the shell are predominant. These ridges are thick but are ultimately much smoother and feature-less at the micro-/nano-scale. 1 ct Euro coin is 16.25 mm in diameter.

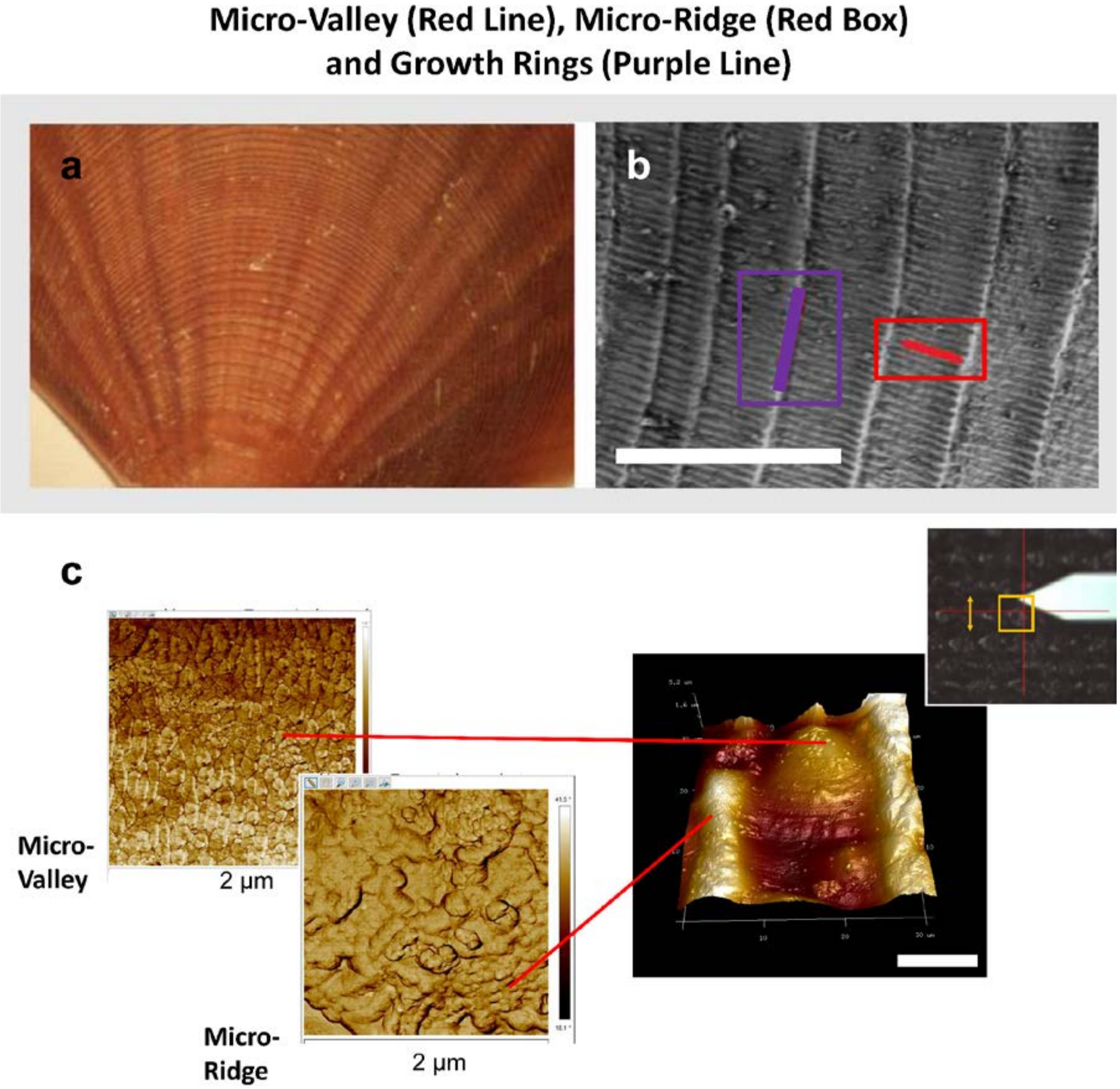

Supplementary Figure 3. Visual photographs alongside high-resolution Atomic-Force Microscopy (AFM) mapping of sub-microscopic features. a) The *A. colbecki* possesses concentric growth rings at the millimetric scale, which separates b) orthogornal micro-ridges that forms dense line patterns (scale bar – 500 μm). Under high resolution AFM mapping, these c) micro-ridges are separated by micro-valleys which are composed of much smoother nano-grain like protrusions. AFM mapping of valley shows a largely smooth (albeit nano-grained) bottom. Scale bar – 10 μm.

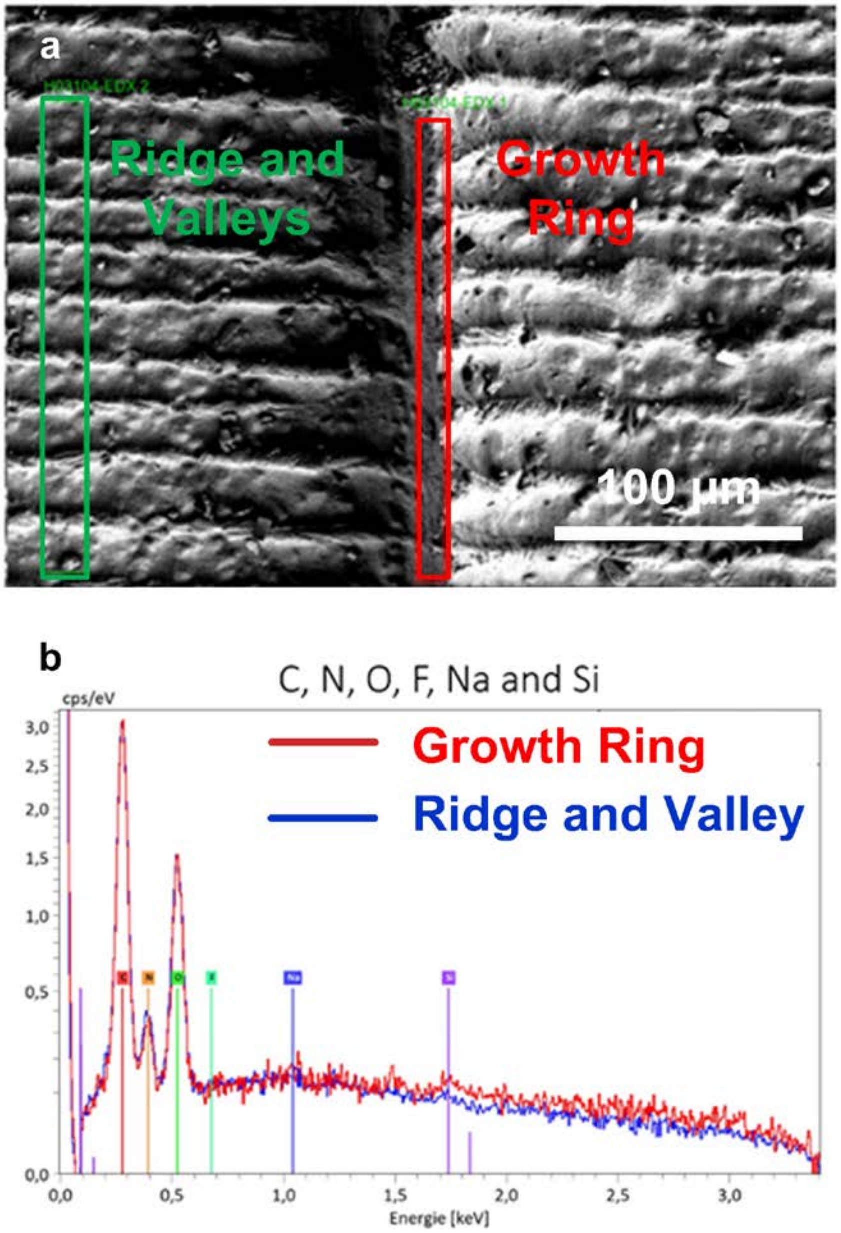

Supplementary Figure 4. Energy-dispersive X-ray spectroscopy (EDS) on shell surface composition. a) SEM of target zones: 1) Growth Ring and 2) Ridge and Valleys. b) Elements: Ca, C, O, F, Na, Mg, Al, Si and Ca tested on both micro-ridges (green box) and growth-ring-peak (red box). Results indicate highly similar surface chemistry properties.

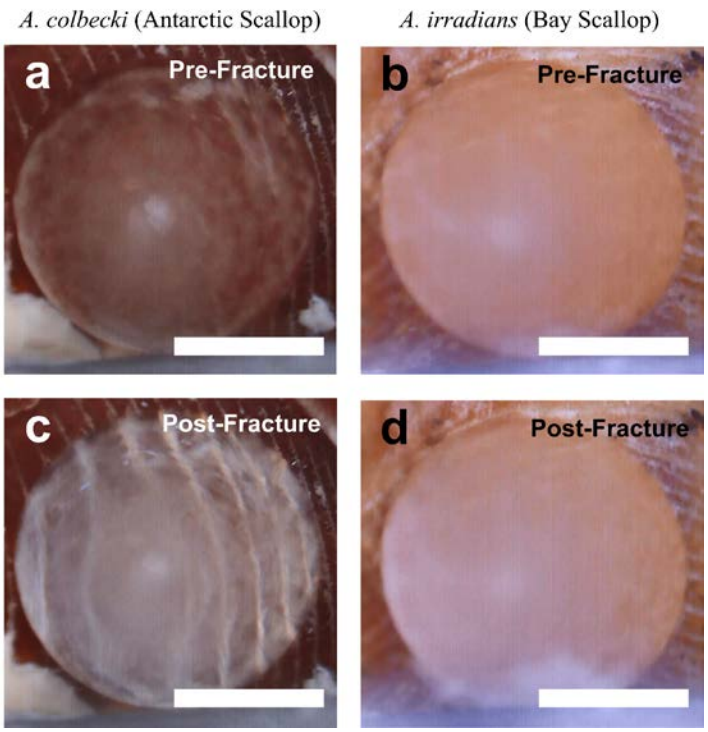

Supplementary Figure 5. Photographic imaging of the Antarctic and Bay scallop shell before and after ice-drop adhesion removal. Force probe (silver-colored block) approaches each frzoen water drop from the bottom, applying a force parallel to the shell surface until the drop detached from the surface. a,c) For the Antarctic scallop, the fracture interface revealed distinctive, visible ice-fracturing lines that follow the contour of the regular, repeating concentric growth rings. b,d) For the bay scallop, the fracture interface was uniform in appearance, as a result of the homogeneity of the shell's surface features. Scale bar – 1 mm.

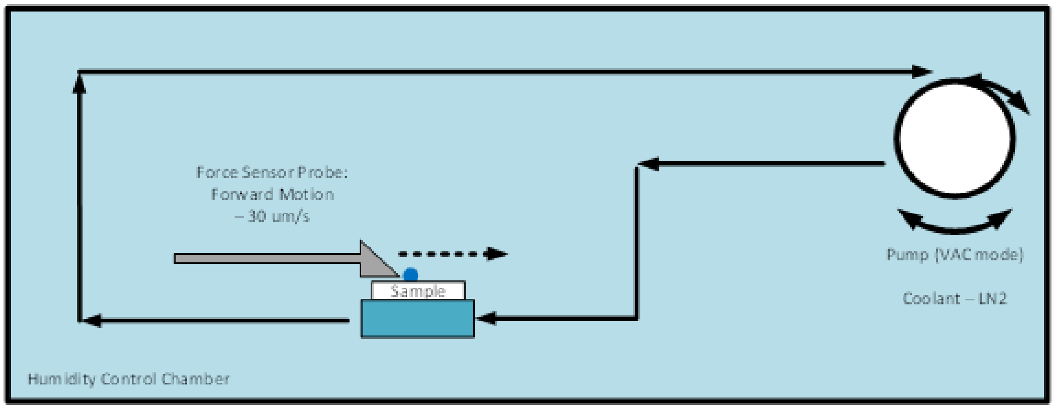

Supplementary Figure 6. Design schematic of the in-air ice adhesion setup.

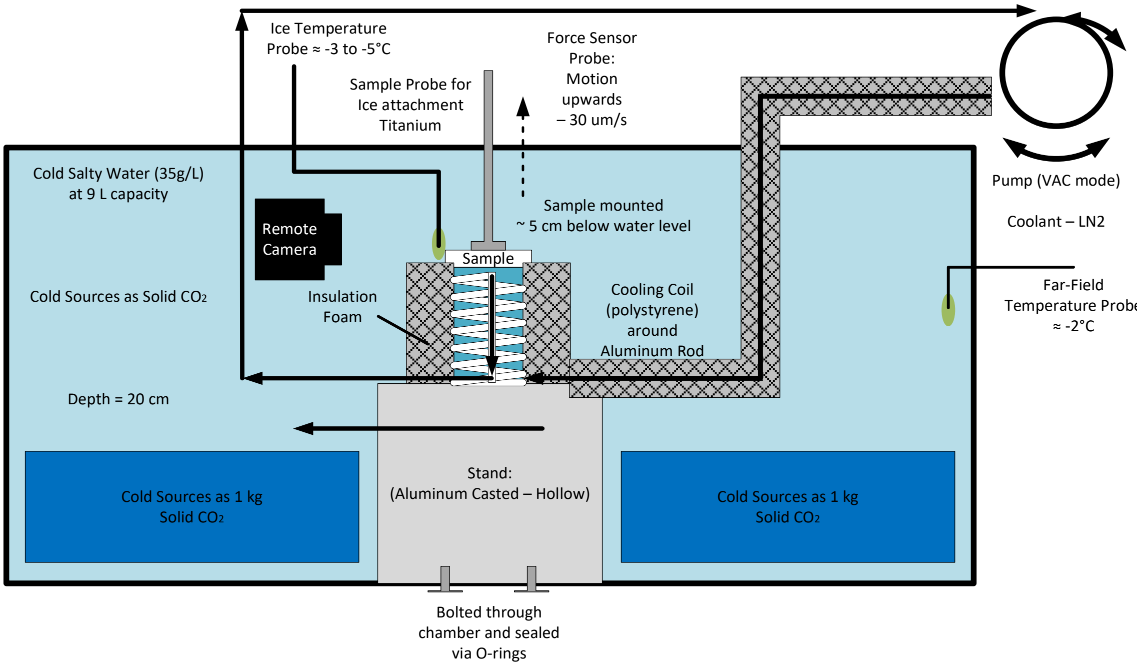

Supplementary Figure 7. Design schematic of the undewater ice adhesion setup.

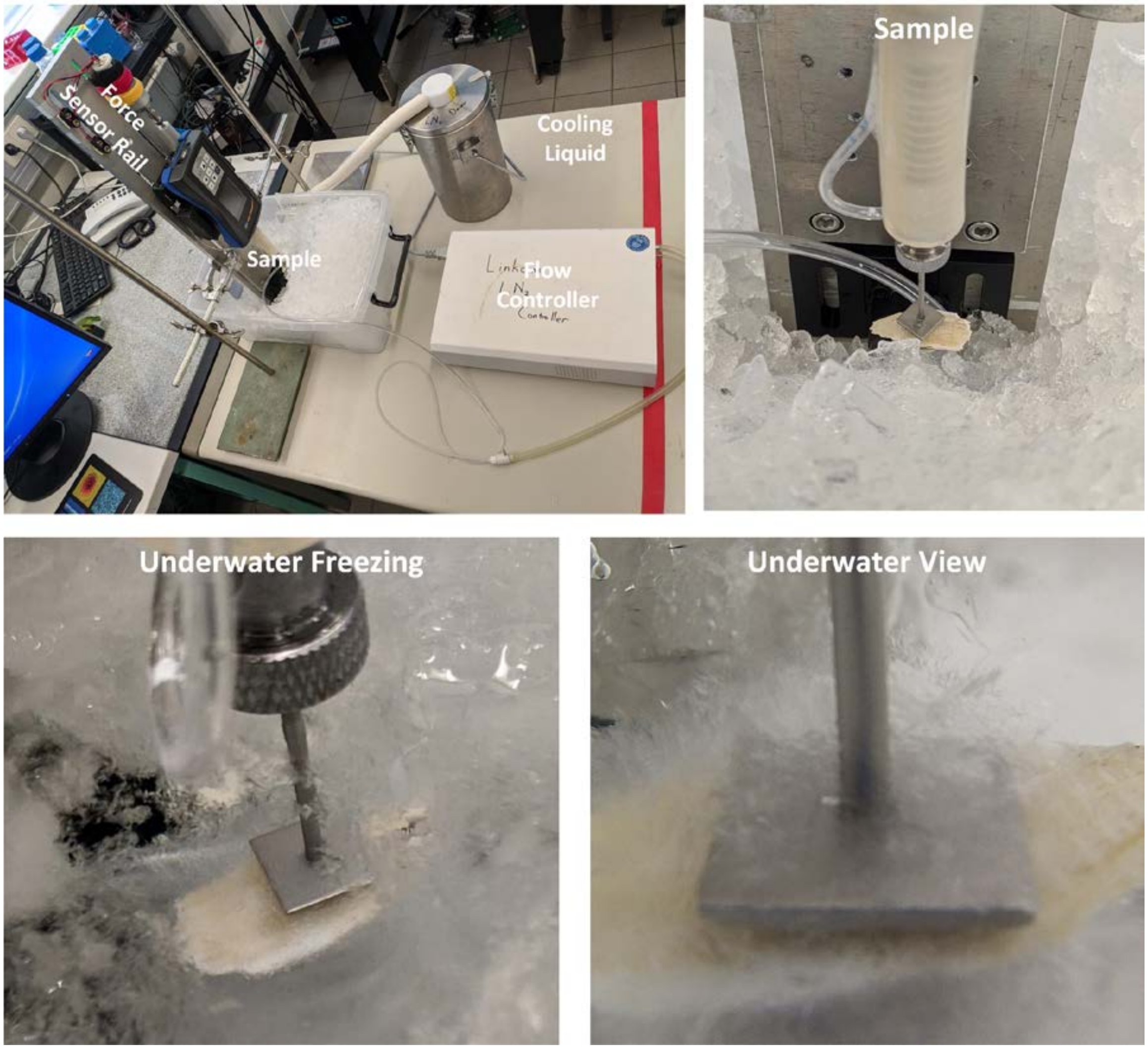

Supplementary Figure 8. Photographs of the undewater ice adhesion setup.

Supplementary References

1 Yuan, Y. & Lee, T. R. in *Surface Science Techniques* (eds Gianangelo Bracco & Bodil Holst) 3-34 (Springer Berlin Heidelberg, 2013).

2 Briggs, D., Chan, H., Hearn, M. J., McBriar, D. I. & Munro, H. S. The Contact Angle of Poly(Methyl Methacrylate) Cast against Glass. *Langmuir* 6, 420-424 (1990).

3 Walker, C. *et al.* Desublimation Frosting on Nanoengineered Surfaces. *ACS Nano* 12, 8288-8296 (2018).

4 Koop, T., Luo, B., Tsias, A. & Peter, T. Water Activity as the Determinant for Homogeneous Ice Nucleation in Aqueous Solutions. *Nature* 406, 611-614 (2000).

5 Qiu, Y., Hudait, A. & Molinero, V. How Size and Aggregation of Ice-Binding Proteins Control Their Ice Nucleation Efficiency. *JACS* 141, 7439-7452 (2019).

6 Heneghan, A. F., Wilson, P. W. & Haymet, A. D. J. Heterogeneous Nucleation of Supercooled Water, and the Effect of an Added Catalyst. *PNAS* 99, 9631-9634 (2002).

7 Dayton Paul, K., Robilliard Gordon, A. & DeVries Arthur, L. Anchor Ice Formation in Mcmurdo Sound, Antarctica, and Its Biological Effects. *Science* 163, 273-274 (1969).

8 Smedley, G. D. *et al.* Molecular Phylogeny of the Pectinoidea (Bivalvia) Indicates Propeamussiidae to Be a Non-Monophyletic Family with One Clade Sister to the Scallops (*Pectinidae*). *Mol. Phylogenet. Evol.* 137, 293-299 (2019).
