# Supplementary material for: Cryofouling avoidance in the Antarctic scallop Adamussium colbecki
Source: Commun Biol. 2022 Jan 21;5:83. doi: 10.1038/s42003-022-03023-6 (PMC8783024; doi:10.1038/s42003-022-03023-6)
Supplement: Supplementary file 2 — Description of Additional Supplementary Files [file 42003_2022_3023_MOESM2_ESM.pdf]

Description of Additional Supplementary Files

File name: Supplementary Movie 1

Description: Live Antarctic scallops in the shallow anchor ice zone.

File name: Supplementary Movie 2

Description: Sponge-colonized Antarctic scallops demonstrate the negative effects of cryofouling.

File name: Supplementary Movie 3

Description: In-air frosting experiments of different scallop shells.

File name: Supplementary Movie 4

Description: Ice adhesion measurements for an Antarctic scallop. Displacement an of ice drop (10 µL) from the surface of shells in a humidity-controlled chamber.

File name: Supplementary Movie 5

Description: Ice adhesion measurements for the Antarctic and Bay scallop. Displacement an of ice drops (10 µL) from the surface of shells.

File name: Supplementary Movie 6

Description: Underwater Ice adhesion measurements for different scallop shells.

File name: Supplementary Movie 7

Description: Underwater Ice adhesion measurements for different scallop shells.

File name: Supplementary Movie 8

Description: In-air frosting experiments with an old Antarctic scallop shell with a damaged surface area.
